# Screening for COVID-19 in Older Adults: Pulse Oximeter vs. Temperature

**DOI:** 10.3389/fmed.2021.660886

**Published:** 2021-04-14

**Authors:** Catherine R. Van Son, Deborah U. Eti

**Affiliations:** ^1^College of Nursing, Washington State University-Vancouver, Vancouver, WA, United States; ^2^College of Nursing, Washington State University-Spokane, Spokane, WA, United States

**Keywords:** older adults, COVID-19, silent hypoxia, temperature, pulse oximeter, atypical presentation

## Introduction

Public health screening for COVID-19 and its mutations are becoming a routine activity, as we assess the safety of resuming interactions with each another. Control efforts have included social distancing, hygiene, masks, and lockdowns. Where available, testing can confirm exposure to COVID-19. Prior to testing, screening is conducted, typically consisting of assessing one's temperature and asking questions related to symptoms and exposures. However, the efficacy of symptom-based screening (temperature and self-report) for COVID-19 has been called into question in recent studies for both the general population and healthcare workers (1, 2). Older adults are another population in which symptom-based screening for COVID-19 should be questioned.

As the pandemic unfolded, older adults have been hardest hit. The statistics are staggering, with older adults making up 45–80% of all hospitalizations, 53% of intensive care admissions, and 80% of deaths (3, 4). However, the media's tone has been that this was not alarming but expected due to age and comorbidities. This paper offers suggestions to mitigate these statistics.

## Temperature and Older Adults

The presence of fever is a key clinical indicator of infection and inflammation (5). Thus, the initial objective screening for COVID-19 has been using temperature measurements to diagnose the presence of infection. Of the general population, 98% of the COVID-19 patients was found to have a fever, along with other symptoms (6).

Fever is defined as a temperature of 100.4°F (38.0°C) or greater (4). However, studies have found that older adults show a lower core body temperature, described as below 98.6°F (36.4°C), using the standard definition of a fever is a less useful indicator of infection with older adults (7, 8). Other studies have found that baseline temperatures may be as low as 94°F (34.4°C) for older adults (9). In a study of 35,488 participants with a mean age of 52.9 years, the baseline temperatures declined with age (−0.02°C every decade, *p* < 0.001) (10). In a sample of 18,630 (aged 20–98 years) with a mean age 58.0 years with equal numbers of male/female participants, researchers found an average basal oral body temperature of 97.3°F (36.2°C) (11). A study of 2410 hospitalized patients with influenza aged ≥65 years found a lower temperature threshold 99°F (≥37.2°C) and captured 78% of influenza-positive individuals, while the CDC's threshold for a fever 100°F (37.8°C) captured only 57% (12).

Lower baseline temperatures may result in overlooking fevers. In fact, upwards of 30% of older adults with serious infections show a mild or no fever (7, 13). One study found older adults (*N* = 1,318), presented to the emergency department with influenza 2–5 days after symptom onset (14). In other studies, seeking treatment occurred up to 1 week after symptom onset (15, 16). This delay in seeking health care increases their mortality risk (14). Therefore, the objective measure of a temperature and the threshold of 100.4 F as a fever indicator does not provide a sufficient indicator of infection in older adults and may delay the diagnosis and treatment for COVID-19 (15, 16).

## Atypical Presentation of COVID-19 in Older Adults

Similar to a fever, older adults lack other usual signs and symptoms of illness onset or exacerbation. Atypical presentations could just be a change in cognitive status or mobility. COVID-19 symptoms include fatigue, body aches, weakness and an increasing loss of taste and smell (17). Each of these symptoms may be dismissed as a normal part of aging. Other symptoms, such as coughing, or shortness of breath may be normal for existing chronic conditions such as chronic obstructive pulmonary disease (COPD) or congestive heart failure (CHF). Older adults with COVID-19 do show typical symptoms such as shortness of breath, fever, and cough; however, many of them do not (17). Atypical presentations of COVID-19 in older adults include a delay in fever and respiratory symptoms. COVID-19 symptoms may present themselves anywhere from 4–5 to 14 days after exposure, which may be too late for initiating interventions and having positive outcomes (18).

## Silent Hypoxia

In April 2020, an emergency room doctor observed COVID-19 patients without visible signs of dyspnea and a SpO_2_ below 90%. He noticed that these patients had a form of oxygen deprivation, which is difficult to detect, called “silent hypoxia,” despite the patients feeling alert and breathing normally (19).

Asymptomatic hypoxia (AH) or silent hypoxia is becoming more prevalent in the COVID-19 literature and is associated with extremely poor outcomes (20). In many cases, AH is associated with a delay in care as the presence of hypoxemia is not identified in the absence of dyspnea (21). In a study from prehospital first responder data, a higher discrepancy was found between oxygen saturation (SpO_2_) and respiratory rates in COVID-19 Acute Respiratory Failure (ARF) patients compared to earlier non-COVID-19 ARF patients (22). Without an SpO_2_ measurement, normal breathing rates could mask profound hypoxia and make the assessment of severity more difficult in an out-of-hospital setting.

Providers must remain attentive while checking for a 3–5% drop in SpO_2_ after mild activity/ambulation, room air, and the presence of hypoxemia without tachypnea (19, 21). However, these symptoms may not be occurring in a clinical setting but at home. For this, there is a portable device: the pulse oximeter, which may detect “silent hypoxemia” in older adults with COVID-19, to be used at home or in a community senior-living setting (22).

## Pulse Oximeters

Pulse oximeters are a noninvasive and painless device that measures oxygen saturation levels in the blood (22). COVID-19 pandemic studies are finding increasing value in using pulse oximetry devices. Studies include the usefulness of oximeters in low-resource settings and predicting clinical deterioration (23, 24). A study evaluating 22 prognostic models for COVID-19 found peripheral oxygen saturation on room air and age was a predictor of clinical deterioration and mortality. In addition, the authors recommended that oximeters should be used in initial screenings as well as community-based monitoring (24).

Given its potential efficacy for detecting changes in SpO_2_, pulse oximeters should be considered to screen for COVID-19 AH in older adults (25, 26). Oximeters are now available as a small, portable, and inexpensive device that can measure SpO_2_ at home. Smartphone apps are being developed so that oximeter readings can be downloaded (using a Bluetooth connection) to the phone and shared with providers. While inaccurate oxygen saturation readings are possible due to incorrect finger placement, nail polish, cold fingers, anemia, or device quality, pulse oximeters may be a valuable screening device for COVID-19 in acute and non-acute settings (25).

Detecting AH is critical for the prevention of infection progression and initiating treatment. Earlier interventions could help patients avoid highly invasive procedures (i.e., intubation and mechanical ventilation) and improve the allocation of scarce healthcare resources (25). One pulse oximetry study using a cutoff of SpO_2_ of 92% decreased the need for hospitalization for COVID-19 positive patients. Checking their SpO_2_ regularly provided patient reassurance and reduced emergency room visits (26). The absence of shortness of breath in an older adult should not be considered to be a good sign. In these patients, pulse oximetry is an important means to improve COVID-19 outcomes (20).

## COVID-19 Screening and Older Adults

Across the nation, testing continues to be inadequate, and temperature screening remains the primary initial objective assessment for COVID-19. The recognition of atypical presentations of infection and physiological aging changes in older adults requires us to implement additional methods of screening to guide clinical decision making.

The diminished febrile response in older adults is a serious disadvantage and suggests fever thresholds should be decreased (9). The absence of shortness of breath in an older adult with comorbidities should not be considered as a sign of well-being. The poor prognoses of asymptomatic hypoxia underscores the severity of this clinical presentation (20). As the absence of fever does not always rule out the presence of an infection, could the screening for “silent hypoxia” help identify older adults with COVID-19 pneumonia earlier? If so, intervening sooner could potentially decrease mortality rates, before the infection progresses to a point of a fever, and the COVID-19 battle is lost.

Halting the spread of the virus among older adults is a challenge, especially in settings where it may be difficult to quarantine, implement social distancing and encourage cognitively impaired older adults to wear masks (27). As screening is essential; decreasing fever thresholds and adding AH screening via a pulse oximeter to routine vital signs is not an unrealistic nor cost prohibitive goal.

## Final Reflections

Symptom-based screening for COVID-19 is a less than precise endeavor, and data being collected during this pandemic is finding that temperature and self-report of exposure and/or symptoms are missing more than 50% of infected individuals (28). Research is needed to determine the most appropriate screening assessments for various infectious diseases and the cohorts exhibiting variations from standard physiological norms.

Clinical presentations and physiological differences in older adults should compel healthcare providers to reconsider current assessment and treatment algorithms. For our most diverse population with considerable variations in illness presentations and disease courses, more appropriate and faster clinical decision making is required. No assumptions should be made that a poor prognosis is part of aging when improvements in public health screening may be achieved and the mortality rate of COVID-19 may be reduced or eliminated.

## Author Contributions

CV developed the concept of the article and wrote the manuscript. DE consulted on content and edited the manuscript.

## Conflict of Interest

The authors declare that the research was conducted in the absence of any commercial or financial relationships that could be construed as a potential conflict of interest.
